# A comparison of diet quality indices in a nationally representative cross-sectional study of Iranian households

**DOI:** 10.1186/s12937-020-00646-5

**Published:** 2020-12-05

**Authors:** Sara Ebrahimi, Sarah A. McNaughton, Rebecca M. Leech, Morteza Abdollahi, Anahita Houshiarrad, Katherine M. Livingstone

**Affiliations:** 1 Institute for Physical Activity and Nutrition, School of Exercise and Nutrition Sciences, Deakin University, Geelong, VIC 3220 Australia; 2 Institute for Physical Activity and Nutrition, School of Exercise and Nutrition Sciences, Deakin University, Melbourne Burwood Campus, 221 Burwood Highway, Burwood, Victoria 3125 Australia; 3 Social Determinants of Health Research Center, Shahid Beheshti University of Medical Sciences, Tehran, Iran; 4 Department of Nutrition Research, National Nutrition and Food Technology Research Institute, School of Nutrition Sciences and Food Technology, Shahid Beheshti University of Medical Sciences, Tehran, Iran

**Keywords:** Dietary patterns, Diet quality, Household, Nutrient adequacy, Sociodemographics, Iran, Healthy eating index, Diet quality index-international

## Abstract

**Background:**

Iranian diet quality has been evaluated using indices that have not been created based on Iranian dietary guidelines. This study aimed to examine the applicability of two diet quality indices by examining their associations with nutrient adequacy, nutrient intakes and sociodemographics.

**Methods:**

Dietary data were collected using three 24-h dietary recalls from Iranian households. Nutrient adequacy was assessed using World Health Organization/Food and Agriculture Organization 2002 (WHO/FAO) cut points. Household diet quality was calculated using the Healthy Eating Index (HEI) and Diet Quality Index-International (DQI-I). Sociodemographics of the household members were assessed. Regression analyses were used to examine associations between diet quality and nutrient adequacy, and between sociodemographics and diet quality.

**Results:**

A total of 6935 households were included in the analysis. Higher household diet quality was associated with adequate intake of calcium (HEI: OR 1.11, 95% CI: 1.10, 1.13; DQI-I: OR 1.14, 95% CI: 1.13, 1.16), vitamin C (HEI: OR 1.19, 95% CI: 1.17, 1.20; DQI-I: OR 1.12, 95% CI: 1.11, 1.12) and protein (HEI: OR 1.01, 95% CI: 1.00, 1.02; DQI-I: OR 1.09, 95% CI: 1.08, 1.09)**.** Higher household diet quality was associated with household heads who were older (> 56 years old) (HEI: β 2.06, 95% CI: 1.63, 2.50; DQI-I β 2.90, 95% CI: 2.34, 3.45), higher educated (college/university completed) (HEI: β 4.54, 95% CI: 4.02, 5.06; DQI-I: β 2.11, 95% CI: 1.45, 2.77) and living in urban areas (HEI: β 2.85, 95% CI: 2.54, 3.16; DQI-I: β 0.72, 95% CI: 0.32, 1.12).

**Conclusions:**

Based on associations with nutrient adequacy and sociodemographics, the applicability of two diet quality indices for assessing the diet quality of Iranian households was demonstrated. Results also indicated DQI-I may be more applicable than HEI for evaluating Iranian nutrient adequacy. Findings have implications for the design and assessment of diet quality in Iranian populations. Future research should examine the link between these diet quality indices and health outcomes.

**Supplementary Information:**

The online version contains supplementary material available at 10.1186/s12937-020-00646-5.

## Background

Chronic diseases, such as cardiovascular diseases and diabetes, are increasing globally and in Iran [1, 2]. As diet plays a critical role in the prevention of chronic disease, it is fundamental to examine the relationship between diet and health outcomes in Iranians [3]. Diets consist of different combinations of foods and nutrients [4]. Dietary patterns approaches consider the amounts and combinations of foods and nutrients consumed and so are being increasingly used [4, 5]. Diet quality indices, which commonly evaluate consumption of key foods and nutrients in accordance with national dietary guidelines (e.g. the Dietary Guidelines for Americans [6] and the Australian Dietary Guidelines [7]), are useful for understanding dietary patterns in the Iranian population [8, 9]. Their usefulness can be assessed by examining their associations with nutrient intakes, biomarkers and sociodemographic characteristics [10].

Previous research has used the Healthy Eating Index (HEI) and Diet Quality Index International (DQI-I) to evaluate Iranian diet quality [11–19]. The HEI was developed to evaluate the adherence of individuals to the United States Department of Agriculture Food Guide Pyramid food and nutrient recommendations [20]. Previous studies in American adults have demonstrated associations between HEI and obesity and chronic diseases, including diabetes and cardiovascular diseases [21–23]. The HEI has been commonly used to evaluate Iranian diet quality [11–18]. The Diet Quality Index-International (DQI-I) was created based on international dietary guidelines to enable cross-cultural diet quality comparisons [24–26] and has been applied to evaluate diet quality in various populations [27–29]. Previous studies in European adults have reported an inverse association between DQI-I and cancer mortality and cardiovascular risk factors, including lipid biomarkers and obesity [27, 30]. However, the DQI-I has only been used in a limited number of Iranian studies [17–19].

Research examining the application of HEI and DQI-I in the Iranian population has been mixed and limited by small and female only samples [11, 13, 17–19]. While studies have shown positive associations between HEI and nutrient intakes, such as protein and iron intake, [11, 19] another study has not [13]. Moreover, while a study among Iranian females (*n* = 1036) showed that higher HEI was associated with higher level of education, [13] other studies showed no significant associations between HEI and education [11, 18]. Although a study of 469 Iranian adults reported no significant association between age and DQI-I [19], another study in 230 Iranian women showed an association between age and DQI-I [17]. Evidence for associations between diet quality and nutrient intakes and sociodemographics from Iranian studies has thus been inconsistent and limited by small non-representative samples [11, 13, 17–19]. Moreover, a diet quality index based on Iranian dietary guidelines does not exist, thus most Iranian research to date has applied the HEI to examine diet quality [11–18]. The DQI-I assesses the adequacy, moderation and balance of a diet, thus it may capture aspects of diet quality related to under- and over-nutrition. This is especially relevant in Iran as it has experienced a nutrition transition over the last three decades [31, 32]. However, the DQI-I has been applied to a limited number of Iranian studies [17–19]. Furthermore, no studies in a large representative sample of Iranian adults have examined both HEI and DQI-I to determine which may be more applicable. As a result, there is a need to establish the applicability of existing diet quality indices for use in the Iranian population by comparing their relationships with nutrient adequacy and nutrient intakes and examining how they vary by sociodemographic characteristics. The primary aim of this study was to examine the associations between two a priori measures of dietary patterns (HEI-2015 and DQI-I) and nutrient adequacy and nutrient intakes in the National Comprehensive Study on Household Food Consumption Pattern and Nutritional Status 2001–2003 [33]. Furthermore, a secondary aim was to examine the association between HEI-2015 and DQI-I and sociodemographics. This study will provide insights into which indices are applicable for use in Iranian nutrition research and will help to understand the characteristics of Iranians with poor diet quality as target groups for future dietary interventions.

## Methods

### Subjects and study design

This study analyzed data from the cross-sectional National Comprehensive Study on Household Food Consumption Pattern and Nutritional Status 2001–2003. Ethics for the study was approved by the Ethics Committee of Shahid Beheshti University of Medical Sciences and was exempted by Deakin University (reference number 2019–288). A cluster sampling method was used to recruit 7248 households from March 2001 to November 2003 from 28 provinces of Iran. Information on sociodemographic characteristics of every member in the household and dietary intakes of the households were collected by interviewers who were trained nutritionists working in the health sector [33]. This manuscript is reported according to the Strengthening the Reporting of Observational Studies in Epidemiology—Nutritional Epidemiology (STROBE-Nut) reporting checklist (Additional File 1) [34].

### Study measures

#### Dietary intakes

Food and nutrient intakes of each household were assessed using three 24-h dietary recalls collected over consecutive days. Households were included in the survey if they had complete data for 2 days (129 households) or 3 days (7119 households) of these 24-h dietary recalls. Trained nutritionists administered the 24-h recalls by interviewing households and checked the questionnaires for completeness. The interviewees were household members who were responsible for the preparation and cooking of meals. Dietary data were not collected during Ramadan and New Year public holiday period [33]. Low and middle-income countries, such as Iran, often collect dietary data at the household level, rather than for each individual, due to the high cost and complexity of collecting individual-level dietary data at a population level [35–37]. As a result, dietary intakes were collected at the household level and were not available for individuals. This study applied the per capita approach, which assumes equal access to intake of food for all members in the household [38]. Foods and drinks consumed by households were recorded as meals and snacks [33]. The dietary data was collected over a whole week including weekdays and weekends and over a period of 1 year for each province to include seasonal variability. The total gram of food intake in each meal was divided by the total number of present persons (including visitors) to calculate the total food intake for each meal. The total food intakes for each meal were then summed up to calculate the total food intake for the day per household.

Food items and nutrient content were coded and evaluated using the Iranian Food Composition Database [33]. A total of 15 nutrients were estimated. For the purpose of this study, sodium (estimated from salt used in cooking), crude fibre, iron, calcium and vitamin C and the percentage of calories from protein and fat were included as they are components of both the HEI-2015 and DQI-I. The Iranian dietary guidelines are food-based guidelines and do not include recommendations for nutrient adequacy. As a result, the FAO cut offs for nutrient adequacy are commonly used in Iranian research [39, 40]. The mean daily requirement of each nutrient per capita in each household was calculated using World Health Organization/Food and Agriculture Organization 2002 (WHO/FAO) recommendations [33]. The average daily household requirements for protein, calcium, iron and vitamin C, were calculated by dividing the total daily requirements of all the household members by the number of household members. Households were categorized into adequate and inadequate groups based on meeting the daily requirements.

#### A priori dietary patterns

HEI-2015 and DQI-I were calculated for every household from an average of the 24-h dietary recalls. The original HEI-2015 comprises 13 components [41]. Of these 13 components, nine components assess adequacy (total and whole fruits, total vegetables, green and beans, whole grains, dairy products, total protein foods, seafood and plant proteins, the proportion of unsaturated fatty acids/saturated fatty acids (SFA), and four components assess moderation (refined grains, sodium, added sugars and SFA). Each item was scored from 0 to 10. The cut points used in the present study were adapted from the original HEI-2015 from Krebs-Smith et al. [41] and have been applied in previous research in Iranians [12–15]. The HEI-2015 was adapted to include eleven components (Additional file 2). Components for the ratio of unsaturated fatty acids to SFA and for SFA were excluded due to a lack of available data. The adapted total score ranged from 0 to 80, with a higher score indicating a higher diet quality.

The original DQI-I includes four main components reflecting variety, adequacy, moderation, and balance [29]. Variety includes two items that assess food group variety and protein source variety (score ranging from 0 to 20). Food group variety includes meat, poultry, fish, eggs, dairy products, beans, grains, fruits and vegetables. Protein source variety comprises meat, poultry, fish, dairy products, beans and eggs. Adequacy includes vegetables, fruits, grains, fibre, protein, iron, calcium, and vitamin C (score ranging from 0 to 40). Moderation consists of total fat, SFA, cholesterol, sodium, empty calorie foods (score ranging from 0 to 30). Balance comprises the ratio of macronutrient ratio and fatty acid ratio (score ranging from 0 to 10). The cut off points used in the present analysis were adapted from the DQI-I from Kim et al. [29] and have been applied in previous Iranian research [17–19]. The DQI-I was adapted to include fourteen measured components (shown in Additional file 3). Components including SFA, cholesterol and fatty acid ratio were excluded in the present analysis due to lack of available food composition data. The adapted total score ranged from 0 to 84, with a higher score reflecting a higher diet quality. The HEI is energy adjusted and is thus applicable to individuals and households [41]. The DQI-I is designed for individuals and is based on age- and sex-specific recommend intakes, although it has been applied to research using household data [29].

#### Sociodemographic characteristics

Information on age, sex, education, household size, area of residence and education were collected for each individual in the household. The interviewees were asked to provide information about each family member. Household level demographic variables were created for the household head. Age of the household head was categorized into three groups including 18–36 years (young adults), > 36–56 years (middle-aged adults), > 56 years (older adults) [42]. Education level of the household head was categorized into three levels based on the total number of the years of education: low (0–5 years; equivalent to completed primary school), moderate (6–12 years; equivalent to completed secondary school), and high (more than 12 years; equivalent to university education). Household size was categorized into less than five members and five members or more based on the 1996 Iranian census - the latest census in Iran at the time of the survey [43].

### Data analysis

Data analyses were performed using Stata (version 16.0; StataCorp). A *P* value of < 0.05 was considered as statistically significant. The number and percentage of participants were reported for all categorical variables (sociodemographic characteristics) and means and standard deviation were reported for continuous variables (dietary intakes). The assessment of which diet quality index was more applicable in the Iranian population was based on which index was associated with adequate intake of more nutrients and a wider range of socio-demographic characteristics, as well as the strength of the associations. To compare the applicability of two diet quality indices for assessing nutrient adequacy and intakes, logistic regression analysis was used to examine the association between diet quality indices (independent variables) and nutrient adequacy (dependent variable). Linear regression analysis was also used to examine the association between diet quality indices (independent variable) and nutrient intakes (dependent variables). To further compare the applicability of two diet quality indices, multiple linear regression analysis was used to assess how diet quality indices (dependent variable) varied across the following sociodemographic characteristics (independent variables): the age of household heads (categorical), sex of household heads (binary), education level of household heads (categorical), area of residence (binary), and household size (categorical). As age, sex, education and area of residence are all known to influence dietary intakes [44–46], all sociodemographic characteristics were included in the same model and models were tested for multi-collinearity by assessing variance inflation factors. Furthermore, as data for the present study were collected using a cluster sampling method by province and households, the sociodemographic characteristics were analysed at the household level to account for potential clustering within households. A complete case analysis approach was used to handle missing data for dependent, independent variables and covariates.

## Results

**Fig. 1:**
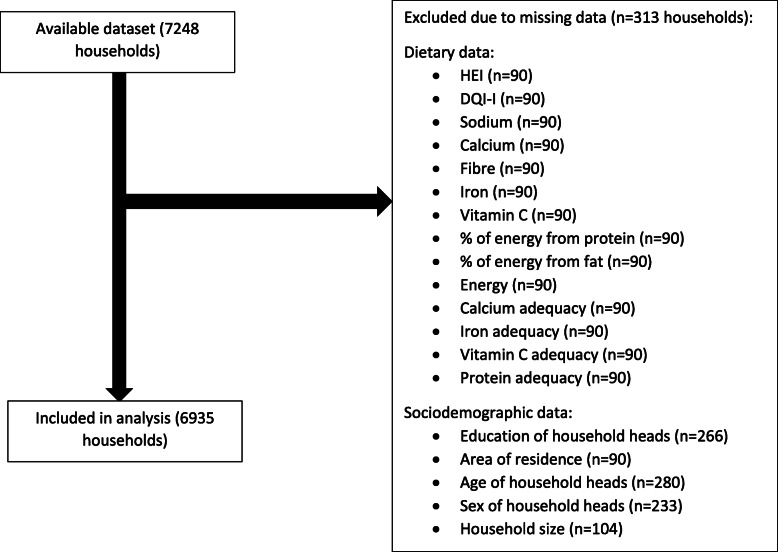
Participant flow diagram

Of the 7248 households who participated in the study (including 129 households with two complete dietary recalls), 313 households were excluded for missing data and a total of 6935 households were included in the present analysis. (Fig. 1) The overall household characteristics and scores for HEI and DQI-I by these characteristics are shown in Table 1. The majority of household heads were middle aged (51%), with a mean age of 46 years (SD 13.3), and 95% of household heads were male. Most household heads had low education (57%) and 65% of households lived in urban areas. The majority of households had five or more family members (54%).

**Table 1:** Sociodemographic characteristics of households and mean HEI and DQI-I scores according to sociodemographic characteristics (*n* = 6935)

Characteristics	Overall N (%)	HEI Mean (SD)	DQI-I Mean (SD)
**Age of household head**^a^			
Young age (reference)	1903 (27.4)	33.4 ± 6.47	36.3 ± 7.85
Middle-aged	3502 (50.5)	34.2 ± 6.42	37.9 ± 7.58
Older	1530 (22.0)	34.0 ± 6.51	39.0 ± 7.70
**Sex of household head**			
Males (reference)	6601 (95.2)	34.0 ± 6.45	37.7 ± 7.75
Females	334 (4.8)	33.0 ± 6.52	37.1 ± 7.44
**Level of education of household head**^b^			
Low (reference)	3929 (56.7)	32.5 ± 5.98	37.6 ± 7.35
Moderate	2341 (33.8)	35.3 ± 6.40	37.4 ± 8.00
High	665 (9.6)	37.9 ± 6.73	39.4 ± 8.81
**Area of residence**			
Rural (reference)	2417 (34.9)	31.4 ± 5.92	37.1 ± 7.34
Urban	4518 (65.2)	35.3 ± 6.34	38.1 ± 7.92
**Household size**			
Less than 5 members (reference)	3218 (46.4)	34.7 ± 6.66	37.4 ± 8.12
5 or more members	3717 (53.6)	33.3 ± 6.21	38.0 ± 7.39

*Abbreviations*: *HEI* Healthy Eating Index, *DQI-I* Diet quality index international

^a^Young age (18–36 years old), middle aged (> 36–56 years old), older (> 56 years old)

^b^low (no formal schooling, less than primary school, the primary school completed), medium (secondary school completed, high school completed), and high (college/university completed)

The associations between diet quality indices and nutrient adequacy are shown in Table 2. The majority of households had inadequate intakes of iron (98%) and calcium (93%). Most households had adequate intakes for protein (78%) and vitamin C (58%). Households with higher HEI and DQI-I scores were more likely to meet the WHO/FAO recommendations for calcium, vitamin C and protein. Households with higher DQI-I scores were also more likely to have adequate intake of iron. With regards to nutrient intakes, households with higher HEI and DQI-I had lower sodium intakes and, higher intakes of fibre, iron, calcium and vitamin C, and protein. Higher fat intake was associated with higher HEI and lower DQI-I (Table 3).

**Table 2:** Logistic regression analysis of the associations between diet quality indices and nutrient adequacy in Iranian households (*n* = 6935)

Nutrients^a^	Overall N (%)	HEI			DQI-I		
		OR	95% CI	***P***-value^b^	OR	95% CI	***P***-value^b^
**Protein (g/day)**							
Inadequate	1549 (22.3)	1.00	-	-	1.00	-	-
Adequate	5386 (77.7)	1.01	1.00, 1.02	0.046	1.09	1.08, 1.09	< 0.001
**Calcium (mg/day)**							
Inadequate	6480 (93.4)	1.00	-	-	1.00	-	-
Adequate	455 (6.6)	1.11	1.10, 1.13	< 0.001	1.14	1.13, 1.16	< 0.001
**Iron (mg/day)**							
Inadequate	6815 (98.4)	1.00	-	-	1.00	-	-
Adequate	120 (1.7)	0.99	0.97, 1.02	0.733	1.08	1.06, 1.11	< 0.001
**Vitamin C (mg/day)**							
Inadequate	2932 (42.3)	1.00	-	-	1.00	-	-
Adequate	4003 (57.7)	1.19	1.17, 1.20	< 0.001	1.12	1.11, 1.12	< 0.001

*Abbreviations*: *HEI* Healthy Eating Index, *DQI-I* Diet quality index international

^a^The average daily household requirements were calculated by dividing the total daily requirements of all the household members by the number of household members

^b^Associations between diet quality indices (continuous independent) and nutrient adequacy (categorical dependent) were examined by using Wald tests of associations for logistic regression

**Table 3:** Linear regression analysis of the association between diet quality indices and nutrient intakes in Iranian households (*n* = 6935)

Nutrients	Overall Mean (SD)	HEI			DQI-I		
		β - Coeff	95% CI	***P***-value^a^	β - Coeff	95% CI	***P***-value^a^
**Energy (kcal/day)**	2636 (695)						
**Protein (% energy)**	11.0 (1.75)	0.08	0.07, 0.09	< 0.001	0.09	0.08, 0.09	< 0.001
**Fat (% energy)**	24.6 (7.49)	0.26	0.23, 0.29	< 0.001	− 0.30	− 0.32, − 0.28	< 0.001
**Sodium (mg/day)**	2264 (2566)	− 80.8	− 90.0, − 71.7	< 0.001	− 42.1	− 49.9, − 34.4	< 0.001
**Fibre (g/day)**	11.9 (4.92)	0.13	0.11, 0.15	< 0.001	0.31	0.29, 0.32	< 0.001
**Iron (mg/day)**	15.0 (5.02)	0.04	0.02, 0.06	< 0.001	0.32	0.31, 0.33	< 0.001
**Calcium (mg/day)**	594 (247)	12.5	11.6, 13.4	< 0.001	17.1	16.5, 17.7	< 0.001
**Vitamin C (mg/day)**	62.2 (49.3)	3.67	3.51, 3.82	< 0.001	2.9	2.8, 3.1	< 0.001

*Abbreviations*: *HEI* Healthy Eating Index, *DQI-I*; Diet quality index international

^a^Associations between diet quality indices (continuous independent) and nutrient intakes (continuous dependent) were examined by using Wald tests of associations for linear regression

The associations between diet quality indices and sociodemographic characteristics are summarized in Table 4. Households where the household heads were older and more highly educated had higher HEI and DQI-I scores. Households with female household heads had lower HEI scores, while higher HEI and DQI-I scores were observed in households living in urban areas. Higher HEI scores, but not DQI-I, were associated with larger household sizes (Table 4).

**Table 4:** Multiple linear regression analysis of the associations between sociodemographics and diet quality indices in Iranian households (*n* = 6935)

Characteristics	HEI			DQI-I		
	β - Coeff	95% CI	***P***-value^c^	β - Coeff	95% CI	***P***-value^c^
**Age of household head**^a^						
Young age (reference)	–	**–**	**–**	**–**	**–**	–
Middle-aged	1.49	1.12, 1.86	< 0.001	1.45	0.97, 1.92	< 0.001
Older	2.06	1.63, 2.50	< 0.001	2.90	2.34, 3.45	< 0.001
**Sex of household head**						
Males (reference)	–	**–**	**–**	**–**	**–**	**–**
Females	−0.89	−1.56, −0.21	0.010	−0.86	**−**1.76, − 0.01	0.052
**Level of education of household head**^b^						
Low (reference)	–	–	–	–	–	–
Moderate	2.40	2.07, 2.74	< 0.001	0.39	−0.04, 0.82	0.077
High	4.54	4.02, 5.06	< 0.001	2.11	1.45, 2.77	< 0.001
**Area of residence**						
Rural (reference)	–	–	–	–	–	–
Urban	2.85	2.54, 3.16	< 0.001	0.72	0.32, 1.12	< 0.001
**Household size**						
Less than 5 members (reference)	–	–	–	–	–	–
5 or more members	−0.95	−1.29, −0.63	< 0.001	0.38	−0.03, 0.79	0.069

*Abbreviations*: *HEI* Healthy Eating Index, *DQI-I* Diet quality index international

^a^Young age (18–36 years old), middle aged (> 36–56 years old), older (> 56 years old)

^b^low (no formal schooling, less than primary school, the primary school completed), medium (secondary school completed, high school completed), and high (college/university completed)

^c^Associations between sociodemographic characteristics (categorical independent) and diet quality indices (continuous dependent) were examined by using Wald tests of associations for linear regression. All sociodemographic characteristics were included in the same model

## Discussion

This study examined the associations of HEI and DQI-I with nutrient adequacy and nutrient intakes and whether they varied by sociodemographic characteristics in Iranian households. As there is currently no diet quality index based on current Iranian dietary guidelines, researchers must rely on existing indices to examine Iranian diet quality. To our knowledge, this was the first study to evaluate the applicability of HEI and DQI-I in a nationally representative Iranian sample. The main findings were that households with higher HEI and DQI-I were more likely to meet nutrient adequacy recommendations for calcium, vitamin C and protein, with DQI-I also associated with adequacy of iron intake. In addition, higher HEI and DQI-I were associated with higher intakes of favorable nutrients, such as calcium, iron, fibre, vitamin C and protein and lower intake of unfavorable nutrients, such as sodium. Furthermore, higher HEI and DQI-I were associated with older age, higher education, and living in an urban area. Thus, while both HEI and DQI-I were found to be appliable for assessing diet quality of Iranians, DQI may be more applicable for assessing nutrient adequacy.

The applicability of the HEI and DQI-I for assessing dietary intakes was evidenced by examining associations with nutrient adequacy and nutrient intakes. Our findings for nutrient adequacy align with research in Iran and many countries in the Middle East, where a high proportion of the population has been identified as not meeting nutrient recommendations for iron and calcium [47, 48]. Although few Iranian studies have assessed the association between diet quality and nutrient adequacy, a study of 819 Iranian adults revealed that adults who did not meet the estimated average requirement for iron had lower HEI [11]. While Azadbakht et al. [11] examined 819 adults from one region of Iran (Tehran), the present study provides a more comprehensive understanding of associations between diet quality and nutrient adequacy. Furthermore, while DQI-I was significantly associated with the adequacy of all nutrients investigated, HEI was not significantly associated with the adequacy of iron intake. As iron intake is an important nutrient to consider in the Iranian nutrition transition [32, 48], these findings warrant further investigation. However, these results may also be due to the household dietary assessment methodology, which may not be able to adequately capture the age- and sex- specific dietary recommendations in the DQI-I [29] and the energy density approach in the HEI [41]. Moreover, the components included differed, as the DQI-I includes nutrients such as calcium, iron, vitamin C, while the HEI does not comprise these nutrients [29, 41]. Consistent with previous research, both indices were positively associated with higher intake of fibre, calcium, iron, vitamin C and protein and lower intake of sodium [11, 19]. As foods and nutrients are not consumed in isolation, the associations between both indices and intake of these nutrients will have been driven by intakes of certain food groups. For example, higher calcium intake may be due to higher intake of dairy products, while higher intake of vitamin C and fibre may be the result of higher intake of fruits and vegetables. However, a positive association was observed between fat intake and HEI. This is likely to be due to a lack of data on fatty acid types, which were indicators of fat intake in the HEI that we were not able to evaluate in this study. Future research should ensure that these dietary components are captured.

Our results showed that households’ age and education are associated with better diet quality are consistent with previous research. Evidence suggests older Iranian adults have higher HEI and DQI-I compared to younger adults [13, 18]. These results may be explained by young adults often having poor dietary behaviours, such as eating discretionary foods outside of the home more often [49]. In line with our results, higher levels of education have been positively associated with adherence to healthier diets in Iranians [50–52]. For example, a study in 1036 Iranian women revealed that higher HEI was associated with higher levels of education [13]. Possible explanations for these findings are that higher education is linked to better understanding of the consequences of an unhealthy diet and that higher education is often linked to higher income and a greater ability to purchase healthy foods [53].

In contrast with our findings, previous studies in Iranian adults have indicated that women have higher HEI [11, 18]. This may be due to different sample characteristics and methodologies. While the present study included Iranian households from 28 provinces of Iran and evaluated the diet quality of household heads in relation to sex, the other studies included between 467 and 819 individuals from Tehran. These studies thus represent a much smaller sample diversity, where women were recruited from urban areas only, in contrast to the present study where participants were included from rural and urban areas of Iran [11, 18]. There are, however, other possible explanations. This study has compared the diet quality of households with female-headed households and male-headed households. Previous research that has examined the socioeconomic position of female-headed households and male-headed households in the National Comprehensive Study on Household Food Consumption Pattern and Nutritional Status 2001–2003 has indicated that the socioeconomic position of female-headed households was lower than male-headed households [54]. Moreover, evidence suggests that households with female household heads have lower socioeconomic position compared to households with male household heads, and a higher socioeconomic position is generally associated with healthier dietary patterns [55, 56]. The findings of the present study indicate that higher HEI, but not DQI-I, was inversely associated with larger household size. This result may be explained by the fact that food access could be more challenging among larger families compared to smaller families [57].

While previous studies in the Iranian population have not investigated the association between diet quality and area of residence, the results from the present study are consistent with similar research. Findings from a large study of Iranian adults indicated that individuals living in provinces with higher rate of urbanization consumed more fruits and vegetables [58]. In addition, a study of 2302 Iranian adolescents showed that those in rural areas consumed more high-calorie, low-nutrient density foods than those in urban areas [59]. Comparable with international studies, a study of 14,584 Chinese individuals showed that the Chinese HEI was higher among urban participants [60]. Our findings illustrated that higher HEI and DQI-I were observed among households in urban areas, which may be explained by rural residents having more difficulty in accessing nutritious foods compared to urban residences [61]. Traveling to food stores and accessing fresh foods is more challenging in rural areas than in urban area, thus rural residents may be more likely to purchase processed foods that store longer and are less nutritious [62, 63].

The present study has several strengths. This study used multiple days of 24-h dietary recall data from a large, nationally representative sample of households from all provinces of Iran conducted by the National Nutrition and Food Technology Research Institute [33]. Dietary data from a nationally representative sample of the population is critical for nutrition policy. As a result, the evidence from these surveys is important for understanding population-level food and nutrient intakes and for informing the design of culturally appropriate healthy eating recommendations [35–37]. The present study applied the HEI and DQI-I to evaluate Iranian diet quality, which include similar components to the Iranian Dietary Guidelines, such as recommendations for increasing fruit and vegetable intake and reducing salt intake [40]. A diet quality score has yet to be designed based on the Iranian Dietary Guidelines, thus these findings are an important basis for future research. The HEI and DQI-I have a number of strengths, including that they both comprise a combination of food groups and nutrients [9]. This provides information for both nutrient and food-based guidelines. Furthermore, these indices include key components that are important to health, such as wholegrains, added sugars, sodium and calcium and dietary variety of specific food groups [29, 41]. In addition, the scoring system of both indices is continuous and proportional, which more effectively reflects the adherence to recommendations compared to cut-off scoring systems [9]. Lastly, the HEI score is density standardized and is thus independent of an individual’s energy intake, making it an appropriate index to evaluate the diet quality of households [41]. Further research is needed to examine how the applicability of DQI-I varies between Iranian individuals and households.

This study had potential limitations. Firstly, dietary intake data were collected at the household level, an approach often used in low and middle-income countries, such as Iran, due to the complexity and high-cost of individual-level dietary surveys [36]. Household dietary data are less precise than individual dietary data because they may not capture food waste and foods consumed outside of the home, and the approach assumes that each household member has consumed the foods relative to his/her energy and nutrient requirements [36]. The per capita approach used in the present study assumes equal distribution of food intakes between household members regardless of age, sex and physical activity [64]. While there is a paucity of research into the validity of household dietary assessment methodologies for estimating individual dietary intake in low and middle-income countries, evidence suggests that the per capita approach may overestimate individual dietary intakes from 24-h dietary recalls [38, 65]. Secondly, while HEI is independent of an individual‘s energy intake [41] and has been used in previous research to assess the diet quality of households [66, 67], the DQI-I was designed to assess the diet quality of individuals [29]. As a result, the DQI-I may be subject to age and sex-specific limitations when applied at a household level. These limitations should be taken into consideration when interpreting the findings and comparing with studies that applied the DQI-I in individuals. Thirdly, there was a lack of information on alcohol consumption in this survey that is a common limitation in studies from Iran and other Middle-East countries [68]. This limitation needs to be considered when comparing results with studies that assessed alcohol intakes. Fourthly, there were limited data on certain nutrients due to gaps in Iranian food composition tables used in cooking, and fibre based on crude, rather than dietary fibre, may not fully represent diet and intakes of certain nutrient (i.e. sodium based on salt intake). As a result, the diet quality indices were adapted to include fewer components, which limits comparisons with other studies. Although these missing nutrients were captured within other components, such as total fat and empty calories, this limitation should be taken into account when comparing results with studies that used the original HEI and DQI-I. Fifthly, data for the present study were collected using a cluster sampling method by province and household. While all variables included in the present analysis were collected at the household level, no information was available to account for potential clustering by province. Lastly, this study analyzed data from an Iranian national survey conducted in 2001–2003. Since Iran has experienced a nutrition transition over the last three decades, in which high quality foods such as fruits, vegetables, and legumes have been replaced by foods high in added sugars, fat and salt and refined grains, [32, 48, 69] our findings should be interpreted with these changes in dietary intake in mind. Nevertheless, these data represent the most recent nationally representative sample of Iranian households and contain detailed dietary data that are appropriate for answering our research question.

## Conclusions

The present study showed that adequate intake of calcium, vitamin C and protein and higher intake of favorable nutrients (e.g. calcium, fibre) and lower intake of un-favorable nutrients (e.g. sodium) were associated with better diet quality (DQI-I and HEI). Furthermore, higher diet quality was associated with older age and higher education of household heads and households living in urban areas. While our findings demonstrate that both diet quality indices are applicable for assessing the overall diet of Iranian households, DQI-I may be more applicable than HEI for assessing nutrient adequacy, including iron. Future research should extend these findings to examine whether better household diet quality is associated with better health outcomes, thus enabling the design of targeted dietary interventions to improve population health.

## Supplementary Information

**Additional file 1: Table S1.** Strengthening the Reporting of Observational Studies in Epidemiology—Nutritional Epidemiology (STROBE-nut). This table describes the STROBE checklist

**Additional file 2: Table S2.** Adapted Healthy Eating Index (HEI-2015) components and standards for scoring. This table describes the scoring of the Healthy HEI-2015 when adapted to this study

**Additional file 3: Table S3.** Adapted Diet Quality Index International (DQI-I) components and standards for scoring. This table describes the scoring of the DQI-I when adapted to this study

## Data Availability

The datasets used in the current study are available from the co-author, Morteza Abdollahi, on reasonable request.

## Abbreviations

DQI-I: Diet Quality Index-International
FAO: Food and Agriculture Organization
HEI: Healthy Eating Index
SFA: Saturated Fatty Acids
WHO: World Health Organization
