# Supplementary material for: A comparison of diet quality indices in a nationally representative cross-sectional study of Iranian households
Source: Nutr J. 2020 Dec 5;19:132. doi: 10.1186/s12937-020-00646-5 (PMC7719237; doi:10.1186/s12937-020-00646-5)
Supplement: Supplementary file 1 — Additional file 1: Table S1. Strengthening the Reporting of Observational Studies in Epidemiology—Nutritional Epidemiology (STROBE-nut). This table describes the STROBE checklist [file 12937_2020_646_MOESM1_ESM.docx]

**Supplementary Table 1.** Strengthening the Reporting of Observational Studies in Epidemiology—Nutritional Epidemiology (STROBE-nut)

| **Item** | **Item nr** | **Reported on page #** |
| --- | --- | --- |
| **Title and abstract** | 1 | 1-3 |
| **Introduction** |  |  |
| Background, rationale | 2 | 4,5 |
| Objectives | 3 | 5,6 |
| **Methods** |  |  |
| Study design | 4 | 6 |
| Settings | 4 | 6 |
| Participants | 5 | 6 |
| Variables | 6 | 6-10 |
| Data sources - measurements | 7 | 6-10 |
| Bias | NA | NA |
| Study Size | 8 | 6 |
| Quantitative variables | 9 | 6-10 |
| Statistical Methods | 10 | 10 |
| **Results** |  |  |
| Participants | 11 | 11 and Figure 1 |
| Descriptive data | 12 | 11 and Table 1 |
| Outcome data | 13 | 11,12 and Tables |
| Main results | 14 | 11,12 and Tables |
| Other analyses | NA | NA |
| **Discussion** |  |  |
| Key results | 15 | 12 |
| Limitation | 16 | 17,18 |
| Interpretation | 17 | 12-16 |
| Generalizability | 18 | 12-18 |
| **Other information** |  |  |
| Funding | 19 | 20 |
| Ethics | 20 | 20 |
| Supplementary material | 21 | Separate document |
