# Supplementary material for: A comparison of diet quality indices in a nationally representative cross-sectional study of Iranian households
Source: Nutr J. 2020 Dec 5;19:132. doi: 10.1186/s12937-020-00646-5 (PMC7719237; doi:10.1186/s12937-020-00646-5)
Supplement: Supplementary file 2 — Additional file 2: Table S2. Adapted Healthy Eating Index (HEI-2015) components and standards for scoring. This table describes the scoring of the Healthy HEI-2015 when adapted to this study [file 12937_2020_646_MOESM2_ESM.docx]

**Supplementary Table 2**. Adapted Healthy Eating Index (HEI-2015) components and standards for scoring^1^

| **Component** | **Indicator and description** | **Score range** | **Criteria for maximum score** | **Criteria for minimum score** |
| --- | --- | --- | --- | --- |
| **Adequacy** |  | 0-40 |  |  |
| Total fruit | Includes fruit juice | 0-5 | ≥ 0.8 cup equiv. per 1000 kcal | No fruit |
| Whole fruit | Includes all forms except juice | 0-5 | ≥ 0.4 cup equiv. per 1000 kcal | No whole fruit |
| Total vegetables | Includes any beans and peas | 0-5 | ≥ 1.1 cup equiv. Per 1000 kcal | No vegetable |
| Greens and beans | Includes any beans and peas, not counted as total protein foods | 0-5 | ≥ 0.2 cup equiv. per 1000 kcal | No dark green vegetable or beans and peas |
| Whole grains | Includes just whole grains | 0-10 | ≥ 1.5 oz equiv. per 1000 kcal | No whole grains |
| Dairy | Includes all milk products such as fluid milk, yogurt, cheese and fortifies soy beverages | 0-10 | ≥ 1.3 cup equiv. per 1000 kcal | No dairy |
| Total protein foods | Beans and peas are included here (and not with vegetables) when the total protein foods standard is otherwise not met | 0-5 | ≥ 2.5 oz equiv. per 1000 kcal | No protein foods |
| Seafood and plant proteins | Includes seafood, nuts, seeds, soy products (other than beverages) as well as beans and peas counted as total protein foods | 0-5 | ≥ 0.8 oz equiv. per 1000 kcal | No seafood or plant proteins |
| **Moderation** |  | 0-40 |  |  |
| Refined grains | Includes any refined grains such as bread, rice, pasta | 0-10 | ≤ 1.8 oz equiv. per 1000 kcal | ≥ 4.3 oz equiv. per 1000 kcal |
| Sodium | Includes all sodium intake | 0-10 | ≤1.1 gram per 1000 kcal | ≥ 2.0 grams per 1000 kcal |
| Added sugars | Calories from added sugars | 0-10 | ≤ 6.5 % of energy | ≥ 26% of energy |

1 The components including ratio of UFA to SFA and saturated fat have been excluded due to lack of available data
