# Supplementary material for: A comparison of diet quality indices in a nationally representative cross-sectional study of Iranian households
Source: Nutr J. 2020 Dec 5;19:132. doi: 10.1186/s12937-020-00646-5 (PMC7719237; doi:10.1186/s12937-020-00646-5)
Supplement: Supplementary file 3 — Additional file 3: Table S3. Adapted Diet Quality Index International (DQI-I) components and standards for scoring. This table describes the scoring of the DQI-I when adapted to this study [file 12937_2020_646_MOESM3_ESM.docx]

**Supplementary Table 3**. Adapted Diet Quality Index International (DQI-I) components and standards for scoring^1^

| **Components** | **Indicator and description** | **Score range** | **Criteria for score range** |
| --- | --- | --- | --- |
| **Variety** |  | 0-20 |  |
| Variety-food groups | meat, poultry, fish, eggs, dairy, beans, grain, fruit, vegetable | 0-15 | ≥ 1 serving from each food group/d=15  Any 1 food group missing/d=12  Any 2 food groups missing/d=9  Any 3 food groups missing/d=6  ≥4 food groups missing/d=3  None from any food groups=0 |
| Variety-protein sources | meat, poultry, fish, dairy, beans, eggs | 0-5 | ≥ 3 different sources/d=5  2 different sources/d=3  From 1 source/d=1  None=0 |
| **Adequacy** |  | 0-40 |  |
| Vegetable group | Includes all type of vegetables | 0-5 | ≥3-5 servings/d=5, 0 servings/d=0  ≥ 100%  < 100-50%  < 50% ^2^ |
| Fruit | Includes all type of fruits | 0-5 | ≥2-4 servings/d=5, 0 servings/d=0  ≥ 100%  < 100-50%  < 50% ^2^ |
| Grain group | Include all type of grains | 0-5 | ≥ 6-11 servings/d=5, 0 servings/d=0  ≥ 100%  < 100-50%  < 50% ^2^ |
| Fibre | Intake of fibre | 0-5 | ≥20-30 g/d=5, 0 g/d=0  ≥ 100%  < 100-50%  < 50% ^3^ |
| Protein | Intake of protein | 0-5 | ≥ 10% of energy/d=5, 0% of energy/d=0  ≥ 100%  < 100-50%  < 50% |
| Iron | Intake of iron | 0-5 | ≥ 100% RDA (AI)/d=5, 0% of RDA AI/d=0  ≥ 100%  < 100-50%  < 50% ^4^ |
| Calcium | Intake of calcium | 0-5 | ≥ 100% AI/d=5, 0% of AI/d=0  ≥ 100%  < 100-50%  < 50% ^4^ |
| Vitamin C | Intake of vitamin c | 0-5 | ≥ 100% RDA (RNI)/d=5, 0% of RDA (RNI) /d=0  ≥ 100%  < 100-50%  < 50% ^4^ |
| **Moderation** |  | 0-18 |  |
| Total fat | Intake of total fat | 0-6 | ≤ 20% of total energy/d=6  >20-30% of total energy/d=3  >30% of total energy/d=0 |
| Sodium | Intake of sodium | 0-6 | ≤ 2400 mg/d=6  >2400-3400 mg/d =3  >3400 mg/d =0 |
| Empty calorie food | Intake of foods that are low in nutrient density  And provides only energy but insufficient nutrients (table sugar, oil and alcohol) | 0-6 | ≤ 3% of total energy/d=6  >3-10% of total energy/d=3  >10% of total energy/d=0 |
| **Overall balance** | macronutrient ratio and fatty acid ratio | 0-6 |  |
| Macronutrient ratio (CHO: protein: fat) ^5^ | The ratio intake of carbohydrate, protein and fat | 0-6 | 55-65: 10 -15: 15-25 =6  52-68: 9-16: 13-27 =4  50-70: 8-17: 12-30 =2  Otherwise=0 |

Abbreviations: CHO; carbohydrates, RDA; recommended dietary allowance, RNI; recommended nutrient intake, AI; Adequate intakes

1 The components including SFA, cholsterol and fatty acid ratio were excluded due to lack of available data

2 Based on three energy levels introduced in the Food Guide Pyramid (1992) 7118 KJ (1700 kcal)/ 9211 (2200 kcal)/ 11340 KJ (2700 kcal) diet; 1 kcal= 4.1868 KJ

3 > 20, 25 and 30 g for three energy levels introduces in the 1992 Food Guide Pyramid, respectively

4 According to dietary reference intake recommendation

5 Ratio of energy from carbohydrate to protein to fat
